# When Collective Knowledge Meets Crowd Knowledge in a Smart City: A Prediction Method Combining Open Data Keyword Analysis and Case-Based Reasoning

**DOI:** 10.1155/2018/7391793

**Published:** 2018-10-03

**Authors:** Ohbyung Kwon, Yun Seon Kim, Namyeon Lee, Yuchul Jung

**Affiliations:** ^1^Professor, School of Management, Kyung Hee University, Seoul, Republic of Korea; ^2^Assistant Professor, Graduate School of Global Development & Entrepreneurship, Handong Global University, Pohang, Republic of Korea; ^3^Assistant Professor, Department of IT Management, Hanshin University, Osan, Republic of Korea; ^4^Assistant Professor, Department of Computer Engineering, Kumoh National Institute of Technology, Gumi, Republic of Korea

## Abstract

One of the significant issues in a smart city is maintaining a healthy environment. To improve the environment, huge amounts of data are gathered, manipulated, analyzed, and utilized, and these data might include noise, uncertainty, or unexpected mistreatment of the data. In some datasets, the class imbalance problem skews the learning performance of the classification algorithms. In this paper, we propose a case-based reasoning method that combines the use of crowd knowledge from open source data and collective knowledge. This method mitigates the class imbalance issues resulting from datasets, which diagnose wellness levels in patients suffering from stress or depression. We investigate effective ways to mitigate class imbalance issues in which the datasets have a higher proportion of one class over another. The results of this proposed hybrid reasoning method, using a combination of crowd knowledge extracted from open source data (i.e., a Google search, or other publicly accessible source) and collective knowledge (i.e., case-based reasoning), were that it performs better than other traditional methods (e.g., SMO, BayesNet, IBk, Logistic, C4.5, and crowd reasoning). We also demonstrate that the use of open source and big data improves the classification performance when used in addition to conventional classification algorithms.

## 1. Introduction

One of the most important ingredients for smart cities is a healthy environment that improves the quality of life and well-being of its city dwellers. Fostering smart, healthy cities is a mission of the WHO (World Health Organization) Healthy Cities project from 1987 (Phase I) and is currently in Phase VI (2014) [1]. The criteria for smart, healthy cities include the ability to gather, manipulate, and utilize huge amounts of data, generated both within and outside designated city to improve the quality of the city dwellers' environment. Based on the analyzed and processed data and smart decision support systems using these data, an individual's health issue can be diagnosed and treated with the most appropriate decisions within a smart healthy city. Currently, the class imbalance problem skews the data analysis and healthcare decision process in smart environments.

The class imbalance problem arising from datasets that include more instances of some classes than others is a challenging issue for improving the quality of IT service using recommendation algorithms, and this problem can lead to classification problems [2, 3, 4]. Recently, class imbalance has been recognized as a crucial problem in machine learning and data mining. This problem is encountered in multiple domains and may have negative effects on the performance of learning methods based on assumption about a balanced distribution of classes [5, 6, 7]. Class imbalance complicates generalizations and causes decreased performance in classifiers, which are machine learning algorithms used for classification purposes.

To address these class imbalance issues, oversampling and undersampling have been studied as solutions [8]. Oversampling increases the minor category data to balance the classes. In this approach, no new information is added through oversampling. This method increases the likelihood of overfitting because it makes exact copies of data in the minority class [9, 10]. Undersampling deletes samples from the major classes [11]. Information loss from deletion jeopardizes the generalizability of the results. As a further restriction, these sampling methods are only applicable for use in two-class tasks [7]. Tasks requiring more than two classes are more complex, and the performance of classification algorithm is consequently lower [12].

Industries that use social media may benefit from research on open source data. In particular, metadata gathered from users of social media can be used for a variety of marketing, medical, and other applications by these industries. Crowd knowledge refers to processes, activities, and resources that are created and deployed by large, often organization-independent user bases [13]. Crowd knowledge is self-evolving, rapidly changing, domain-based knowledge acquired using social media, search engines, or other open source origin [13]. The beauty of crowd knowledge is that it is open sourced and freely accessible, and it can therefore be mined very easily through the Internet using social media, or search tools. Its coverage is broad, as data from all over the world may be collected from anyone who wants to participate. Crowd knowledge can be less biased than knowledge collected from a relatively small set of data sources, such as in studies using case-based reasoning. Because of the lower bias, crowd knowledge has been used in studies of recommendation systems [14, 15] and Q&A systems [16, 17], in which data are collected inexpensively and connectivity is high. Crowd knowledge can be unreliable because participants who provide data have no obligation or responsibility to affirm that the knowledge is correct. Since the intent of users of crowd knowledge cannot be identified in advance, the knowledge is not well structured according to specific uses. These constraints decrease the accuracy of classifications using crowd knowledge.

In this study, we propose a hybrid reasoning approach combining case-based reasoning as a form of collective knowledge and big data search engine as a source of crowd knowledge. Search engines such as Google are open source tools for continuously accumulating data storage tools. It has less biased collective data, good coverage over time, and unbiased data collection methods, which make it less susceptible to the cold-start problem. These characteristics make crowd knowledge from Google's data a good complement to case-based reasoning to solve classification problems. We used an actual dataset from a commercial wellness care service, operating in China and Korea, to demonstrate the performance of this hybrid method. We then compared the proposed method to conventional classification algorithms in terms of accuracy, TP rate, ROC, and AUC.

The paper is organized as follows: Section 2 provides a literature review on case-based reasoning and crowdsourced knowledge. The proposed method is outlined in Section 3. The experiment and results are described in Sections 4 and 5, respectively.  Section 6 concludes and gives areas of future research.

## 2. Related Work

### 2.1. Case-Based Reasoning

During problem-solving, human beings naturally reuse previous knowledge, accessing information about similar past cases for which they have information. In machine learning, systems and methodologies have been developed to solve new problems in ways similar to human problem-solving. Case-based reasoning (CBR) is one such methodology. CBR is the process by which new problems are solved based on past experiences, where problems were solved and the routines for doing so were memorized [18]. CBR is a methodology combining problem-solving and learning.

The CBR approach was introduced by Roger Schank and his students about 37 years ago [19]. In the early 1980s, several models were presented such as Schank's dynamic memory model [20], Janet Kolodner's CYRUS [21], Lockheed's CLAVIER [22], and Michael Lebowitz's IPP [23]. In the 1990s, use of CBR grew widely in various fields. Skalle et al. described the employment of the CBR method in the drilling industry [24]. Bhushan and Hopkinson [25] applied CBR to global searches for reservoir analogues. Combined with database systems, CBR has been used to support the interpretation and classification of new rock samples [26]. The Compaq SMART system was developed and applied using CBR to support the Compaq help desk [27]. Particularly relevant to this study is the fact that many CBR systems have been applied in medical decision making, such as CBR for medical knowledge-based systems [28], CBR in the health sciences [29], CBR for the prognosis and diagnosis of chronic diseases [30], case-based medical diagnosis, development, and experimentation [31], and a distributed CBR tool for medical prognosis [32]. Recently, CBR has been applied in problem-solving complex domains, including planning [33], law [34], e-learning [35], knowledge management [36], image processing [37], and recommender systems [38].

CBR follows a cycle of four steps or processes: retrieve, reuse, revise, and retain [18]. A typical CBR system might look something like this: (1) retrieve the most similar cases from all previous cases relevant to solving the new problem, (2) reuse the knowledge in these retrieved cases, (3) revise previous solutions to fit the new problem, and (4) retain information about the resulting experience for solving new problems in future. Based on these principles and previous studies of CBR, the benefits of using CBR in solving real-world problems are as follows: (1) the use of CBR reduces the effort required for knowledge acquisition, (2) CBR requires less maintenance effort relative to other algorithms, (3) CBR improves problem-solving performance through reuse of previously successful solutions, (4) CBR allows reuse of existing data, (5) CBR allows solutions to improve over time and adapt to changes in the environment, and (6) it is easily accepted by users.

Despite the widespread use and acceptance of the CBR method, without statistically relevant data for backing up the results and facilitating generalization, there is no guarantee that the results of problem-solving using CBR are correct [39]. This well-known problem is called the cold-start problem in information systems, based on computer automation of the data modelling. Obtaining sufficient and appropriate information is an intrinsic problem so that statistically relevant inferences can be made. Although the relevant data may generate a result that solves the problem, without sufficient data to back up the results, no statistically sound claims can be made. In addition, class imbalance, by having the total number of data in some classes more numerous than others, exacerbates the cold-start problem. In this study, the authors examine the potential of crowd knowledge-based methodology to overcome this limitation of CBR.

### 2.2. Class Imbalance

Class imbalance causes and exacerbates the cold-start problem [2, 4]. Class imbalance refers to the problem that some classes have many more instances than others. The class imbalance problem is encountered in a large number of domains. For example, in medical diagnosis, class imbalance is often found. The stakes are high in this field because it is necessary for classifiers to be accurate. The cost of erroneously diagnosing a patient as healthy may exceed that of mistakenly diagnosing a healthy person as sick, because the former error may result in loss of life [7].

Class imbalance is common when the collected knowledge is insufficient because it is new or there is a change in the environment, like a fluctuation in consumer preference. Class imbalance contributes to the cold-start problem, which occurs in situations where decisions or historical data are required but for which no dataset has yet been established. This is a widespread problem in recommender and diagnosis systems. Recommender systems suggest choices, items, and services based on users' interests, and their explicit and implicit preference information is pregathered. The implicit and explicit preferences may be related to other users, item attributes, or contexts. For example, a travel recommender provides decision options for specific users from combined preferences, like explicit rating information (e.g., Kim rates Dokdo 9 out of 10 for tourist spots in Korea), implicit information (e.g., Kwon reserved Dokdo travel for next week), item attributes (e.g., Dokdo is not accessible to everyone), demographic information (e.g., Kim and Kwon are male), weather conditions (e.g., Dokdo is an island and experiences much rain during the summer), and other criteria (e.g., other islands such as Jejudo and Ulleungdo).

Researchers have developed algorithms and techniques to avoid the cold-start problem caused by class imbalance, including memory-based algorithms [40], filtering through hard-clustering [41], simultaneous hard-clustering [42], soft-clustering [43], singular value decomposition [44], inferring item-item similarities [45], probabilistic modelling [46], machine learning [47], and list ranking [48]. These techniques all have both advantages and disadvantages. Other methods to cope with the class imbalance problem with the cold-start problem must be developed.

### 2.3. Crowd Knowledge

Innovative technologies related to the Internet changed the definition of the word “crowd.” Currently, a crowd includes not only a group of people or animals, but also a group of anything, for example, documents, pictures, songs, videos, and web pages. On a Web 2.0 site, users may interact and collaborate with each other in a social web, which is represented by a class of websites and applications, as creators of user-generated content in virtual communities. Examples of Web 2.0 applications include Wikipedia, MySpace, YouTube, Linux, Yahoo! Answers, Flickr, Del.icio.us, Facebook, Twitter, and more. Furthermore, the potential for knowledge sharing today is unmatched in history. Never before have so many creative and knowledgeable people been connected by such an efficient and universal network. The Internet provides an incredible wealth of information and diversity of perspective and fosters a culture of mass participation that sustains a fountain of publicly available content. Millions of humans upload their knowledge online for easy storing, searching, and sharing with others. This content is called “crowd knowledge.” The amount of crowd knowledge available on the web exceeds human control.

Crowd knowledge is an imprecisely defined term. In this paper, crowd knowledge is treated as an extension of collective intelligence. Collective intelligence is a shared or group intelligence acquired from various sources such as collaboration, collective efforts, competitions among many individuals, and machines. It is used to make appropriate decisions in many contexts. Collective intelligence, or crowdsourcing, has continuously grown throughout the history of the Internet. Engelbart [49] introduced the idea of collective intelligence in 1963 when he stated that “the grand challenge is to boost the collective IQ of organizations and of society.” His idea was that a human-machine system involving various technologies could harvest collective knowledge to improve collective learning. Other pioneers of the human-machine model in collective intelligence were Norbert Wiener (cybernetics), Buckminster Fuller (system thinker) [50], and Stewart Brand (first large virtual community on the Internet) [51]. The inventor of the World Wide Web, Tim Berners-Lee, shared his vision about the web: “The Semantic Web is not a separate Web but an extension of the current one, in which information is given well-defined meaning, better enabling computers and people to work in cooperation” [52]. Collective intelligence entails the shift of knowledge and power from the individual to the collective.

Collective intelligence is the result of mass collaboration. It is based on four principles [53]: (1) openness, or allowing the sharing of ideas and intellectual property to everyone, (2) peering, which is to open up the opportunity for users to modify and develop through horizontal organization, (3) sharing of ideas, patent rights, and intellectual property in order to expand markets and bring out products faster, and (4) acting globally with no regard for geographical boundaries, allowing access to new markets, ideas, and technology. In addition to these four principles, crowd knowledge includes the following features: (1) unintended data sources, (2) low-value data, and (3) large volume. Crowd knowledge may be related to relevant searches, but also to unintended or unknown areas. Users of crowd knowledge can obtain search results from the collective intelligence acquired by machine learning in user-intended search areas, and other areas, such as biology, psychology, services, books, social media, games, medicine, advertisements, educations, patents, and jobs. This unintended knowledge may be based on data of low value. The value of such data must be increased to generate meaningful connections.

Crowd knowledge may be gleaned from data of almost infinite volume. According to the CSC's Big Data Infographic and Data Evolution report, the amount of data available in the world size increased dramatically in 2012 (1.2 zettabytes) and will be increased exponentially in 2015 (7.9 zettabytes) and in 2020 (35 zettabytes)—1 zettabyte is 1,000,000,000,000 gigabytes [54]. Knowledge based on data of this volume may increase the relevance of the results and potentially improve our capability of making decisions.

The quality of crowd knowledge depends on the degree of user participation, and the flexibility of knowledge as it evolves and responds to shifts in social trends [13]. One application through which crowd knowledge can be obtained is the Google search engine, the most-used search engine on the World Wide Web, where more than three billion searches are made each day. In this paper, we use the Google search engine for the application representing crowd knowledge because it indexes all web pages in the world, and there is no barrier to uploading knowledge from knowledge providers or to searching for knowledge for knowledge users, the results are sensitive to social trends, and it is cost-efficient as it is free.

Crowd knowledge has been utilized in past studies of recommendation systems [14, 15] and Q&A systems [16, 17] to resolve the cold-start problem. Crowd knowledge mitigates the cold-start problem since it is not limited by previous knowledge, models, or cases. Crowd knowledge may need expert knowledge to be verified as relevant before being accepted by an intelligent system.

In this paper, a novel hybrid method combining crowd and case-based knowledge is developed and tested. This hybrid method generates a model, knowledge, or case from crowd knowledge at the first reasoning instance. Based on this model, knowledge, or case, CBR provides results that accurately reflect changes in crowd knowledge over time.

## 3. Proposed Method

**Figure 1:**
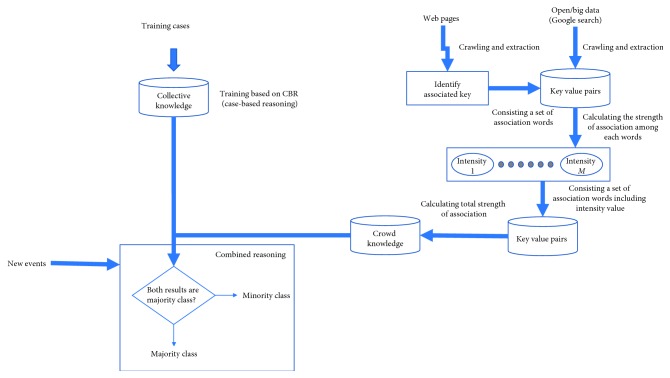
Proposed method.

In this paper, we propose a novel hybrid reasoning method to address the issues with classification involving datasets with class imbalance. To enhance accuracy, the method also involves use of a well-known classification algorithm, CBR, and crowd knowledge. Results of classification systems using crowd knowledge are determined through comparison with features of high relevance to the minority class, and the relation between features of the dataset and the classes. The classification is estimated using open data or big data, which is unstructured and updated continuously. Open data resulting from a Google search is used. As shown in Figure 1, the proposed method consists of three phases: reasoning with crowd knowledge, reasoning with collective knowledge, and combining the results of these two reasoning methods to reach a classification decision.

### 3.1. Reasoning with Crowd Knowledge

The first stage involves mining the crowd knowledge and gathering the associated words from each class from the open source data. To gather the words related to a class word, information regarding URLs or posts is collected by querying “class words” in a Google search or SNS. After downloading web pages identified by a web crawler, or SNS API, such as Twitter4j and Facebook4j, a text analysis is then conducted. The analysis finds synonyms of the class word that can then be queried. A set of associated words is represented as follows:

(1) $$\begin{array}{c} <c_{0},c_{1},\ldots ,c_{m}>, \end{array}$$

where *c*_0_ is the original class word and the rest are synonyms and associated words. The frequency of a word indicates the extent that it is related to the target class word. Words that frequently appear ubiquitously (e.g., *a*, *the*, *person*, and *we*, *and*) are eliminated from the candidate set of associated words. Features in the feature set may also be associated words if the feature set is predefined to gather collective knowledge, as in questionnaires.

Once a set of associated words is identified, the strength of the association is calculated. The strength of association between any two words, *x*_*i*_ and *c*_*j*_, is represented by ∝_*i*,*j*_, as below:

(2) $$\begin{array}{c} \alpha_{i,j}=\frac{\zeta \left(x_{i}⊗c_{j}\right)}{\zeta \left(c_{j}\right)+\zeta \left(x_{i}\right)-\zeta \left(x_{i}⊗c_{j}\right)}, \end{array}$$

where 0 ≤ *α*_*i*,*j*_ ≤ 1, *ζ* is the number of appearances and *a* ⊗ *b* means *a* and *b* appear simultaneously in a given web page. For example, in a Google search, if the frequency of (*insomnia*⊗*depression*) is 209,000 and the frequencies of *insomnia* and *depression* are 907,000 and 3,570,000, respectively; then *α*_*i*,*j*_ is 209,000/(907,000 + 3570,000 − 209,000) = 0.049.

The direction of association is calculated along with the strength of association. The strength of association must be greater than 0, but the direction of association may be positive or negative (+, −). For example, the direction of *insomnia* and *depression* is positive, but the direction of *well*-*being* and *depression* is negative. The direction of association is determined by correlation analysis if collective knowledge exists. Otherwise, the results are gathered through a sentiment analysis, or based on prior knowledge from the acquired URL.

The next step is to calculate the total strength of the association, if more than two associated words are identified in a class. The total strength of the association with every associated word in the class is then calculated. For example, the total strength of the association for *depression* is the average value for the strength of the association in associated words such as *insomnia*, *well-being*, and *tiredness* as follows:

(3) $$\begin{array}{c} \alpha_{i}=\frac{\sum_{\forall i}{\left(-1\right)}^{\beta_{i,j}}\alpha_{i,j}}{\sum_{\forall i}1}, \end{array}$$

where *β*_*i*,*j*_ is a multiplier which is 1 if the direction of the association is negative, or 2 if the direction of the association is positive.

The degree of strength may be added to the concept of class. For example, the concept of class may be a primitive concept (PC, e.g., *depression*), a weak concept (WC, e.g., *weak depression*), a moderate concept (MC, e.g., *regular depression*, *not weak depression*), or a strong concept (SC, e.g., *severe depression*). The total value for the strength of the association (*α*_*i*,*j*,*k*_) is calculated as follows:

(4) $$\begin{array}{c} \alpha_{i,j,k}=\frac{\zeta \left(x_{i}⊗c_{j,k}\right)}{\zeta \left(c_{j,k}\right)+\zeta \left(x_{i}\right)-\zeta \left(x_{i}⊗c_{j,k}\right)}, \end{array}$$

where *k* is an index of the degree adverb that distinguishes the concept of any given associated word as PC, WC, or SC.

Then, *α*_*i*,*j*,*k*_ is identified as the specific level or degree to which a given concept is larger than any other concepts. For example, *insomnia* includes the larger concept combination (*insomnia* and *SC depression*), which allows us to calculate the strength of the association when *insomnia* and *depression* appear simultaneously. The largest concept combination is *insomnia*-*SC depression*, showing the association between *insomnia*-*WC depression* and *insomnia-SC depression*.

The total strength of the association of any given concept is derived as follows:

(5) $$\begin{array}{c} \alpha_{i,\text{con}}=\underset{\forall j\in J_{\text{con}}}{\sum }\overset{K}{\underset{k=1}{\sum }}{\left(-1\right)}^{\beta_{i,j,k}}\frac{\zeta \left(c_{j,k}\right)\times \alpha_{i,j,k}}{\sum_{k=1}^{K}\zeta \left(c_{j,k}\right)}, \end{array}$$

where con is an element in a set of PC, WC, MC, or SC and *J*_con_ is a set of features related to a certain con, which means that it is the set of features observable when the strength of the association in the specific con is greater than that of any other con. The strength of the association is the crowd knowledge of a certain con.

In the proposed method, the reasoning method from the calculated strengths of the association, the subjective average method, and the absolute maximum method are combined. The subjective (or relative) average method distinguishes relatively higher or lower values by comparing the results of the two averages from the inputs, and the strength of the association of the features in the *J*_con_ of the con in a case from a specific sample, and from other cases. The absolute maximum method determines whether the minimum or maximum values of each feature in the *J*_con_ of the con in a case from a specific sample are included within a specific range or if it is compared with other cases. The result is classified as a minority class if one of two results from these methods indicates that the value should be classified as “minor.”

### 3.2. Hybrid Reasoning

Based on the results of reasoning with crowd and collective knowledge, a final classification is made.

To be sensitive to results that designate a minority class, we add the following provision. If at least one result indicates that the case should be classified as minor, the final determination using the combined reasoning method is that the result is the same as those in the minority class. Similarly, when the results obtained using the two reasoning methods determine the case as major, the final determination using the combined reasoning method is that the result is the same as those in the majority class.

## 4. Experiment

To show the feasibility of the idea proposed in this paper, we conducted an experiment. To obtain training and test data, a user survey was conducted prior to the experiment. In the experiment, an actual dataset was archived and used as a training and test set to test the proposed method. The results of the proposed method were then compared with conventional classification methods for performance accuracy.

### 4.1. Procedure

The experiment was conducted according to the method introduced in the previous section. Additional details are provided below.

*Step 1*. First, we collected potentially associated words in a Google search. For example, if the city name is “Seoul,” then we typed “stress temperature Seoul.” As a result, the following words were identified as associated with *stress* and *depression*: *temperature*, *humidity*, *noise*, *illumination*, *anger*, *stress*, *depression*, *fatigue*, *fat*, *blood sugar*, *heart disease*, *hyperlipidemia*, *cancer*, *smoking*, *drinking*, *indigestion*, *insomnia*, *cold*, *allergy*, *neurosis*, *blood pressure*, *diabetes*, *wellness*, *age*, *gender*, *weight*, *solitude*, *hobby*, *yellow dust*, and *inconvenience*.

*Step 2*. Degree words (adjectives) for the words identified in Step 1 were identified from the literature related to stress and depression, as follows: slight—a little, some, succinct, trifling, light, and soft, and serious—not a little, severe, grave, deep, intensive, and intensified.

*Step 3*. Through a Google search, we acquired information as to the frequency of each associated word and degree word. For example, we found the frequency of “*deep depression*,” as well as *depression*. Note that the results for frequency may vary because the data must be collected within a very short time period. For our study, we collected data regarding frequency for all words and all combinations of words in one day. To aid in this process, eight coders were recruited for the experiment.

*Step 4*. The results were divided into two groups: normal and serious. The results for *depression* are displayed as shown in Table 1.

*Step 5*. Based on the results reported in Table 1, we calculated *α*_*i*_, the associated level for each word, using Equation (4). The results are shown in Table 2. For *serious depression*, words such as *noise*, *stress*, *fatigue*, *fat*, *diabetes*, *smoking*, *drinking*, *insomnia*, *allergy*, *blood pressure*, and *wellness* were significantly more often associated with this term than *normal depression*, while *anger*, *cold*, *age*, *gender*, *hobby*, and *yellow dust* were more often associated with *normal depression*. Finally, the pairs of associated words and information about the level of depression or stress were then stored in a crowd knowledge database for later use.

*Step 6*. Once a crowd knowledge database was established, we could predict a person's level of depression or stress by combining the classification results obtained using a collective knowledge method (i.e., CBR) with those obtained from crowd knowledge.

*Step 7*. In this study, to validate our method's performance, we utilize data obtained from a public well-being life care service for a city about the stress and depression levels of Korean citizens. This dataset was constructed in 2014. All data were gathered as part of company-driven wellness life care application and were provided by customers who actually or may potentially use the commercial system for treatment. We collected 334 valid (i.e., responses which meet the questionnaire restrictions) data in total. Descriptive statistics relating to the subjects' profiles are summarized in Table 3. The class imbalance of the datasets is shown in Table 4. In the public health system, the users were requested to estimate the degree of stress and depression they were experiencing on a 7-point Likert scale (1: I feel no stress/depression now, 7: I feel serious stress/depression now).  Table 5 is the questionnaire we used for the survey, and Table 6 is the raw data samples from the survey. Scores were then categorized as follows: 1–5 = normal, 6 or 7 = abnormal.  Table 7 shows comparing the degree of stress and depression scores before and after categorization. Respondents with scores greater than or equal to 6 were advised to consult a doctor. As shown in Table 4, most of the subjects in this study scored within the range of normality (tagged as 1).

### 4.2. Results

To analyze the performance of the proposed method, the results were compared with those from the traditional methods of measuring performance, such as overall accuracy, TP (true positive) rate, FP (false positive) rate, precision, and recall. TP and TN (true negative) are the number of positive and negative samples that are classified correctly. FN (false negative) and FP are the number of misclassified positive and negative samples, respectively.  Table 8 displays a confusion matrix. Equations (6) through (10) outline our measures of performance:

(6) $$\begin{array}{c} \text{accuracy}=\frac{\left(\text{TP}+\text{TN}\right)}{\left(\text{TP}+\text{FN}+\text{FP}+\text{TN}\right)}, \end{array}$$

(7) $$\begin{array}{c} \mathrm{TP}\text{rate}=\frac{\text{TP}}{\left(\text{TP}+\text{FN}\right)}, \end{array}$$

(8) $$\begin{array}{c} \mathrm{FP}\text{rate}=\frac{\text{FP}}{\left(\text{FP}+\text{TN}\right)}, \end{array}$$

(9) $$\begin{array}{c} \text{precision}=\frac{\text{TP}}{\left(\text{TP}+\text{FP}\right)}, \end{array}$$

(10) $$\begin{array}{c} \text{recall}=\frac{\text{TP}}{\left(\text{TP}+\text{FN}\right)}. \end{array}$$

However, the performance outcomes in Equations (6)–(10) cannot fully be compared when there is class imbalance in the dataset. In this situation, accuracy is no longer a comparable measure, since it does not distinguish between the dataset numbers of correctly classified examples (TPs) of different classes. To achieve comparable results for both classes, ROC (the receiver operating characteristic) analysis and AUC (area under the ROC) curve are used [56]. The AUC measure is computed as follows:

(11) $$\begin{array}{c} \text{AUC}=\underset{i}{\sum }\left(\left({\text{TP}}_{{\text{rate}}_{i}}\cdot \Delta {\text{FP}}_{\text{rate}}\right)+\frac{1}{2}\left(\Delta {\text{TP}}_{{\text{rate}}_{i}}\cdot \Delta {\text{FP}}_{\text{rate}}\right)\right). \end{array}$$

The ROC curve is a two-dimensional graph in which the TP rate is plotted on the *y*-axis and the FP rate is plotted on the *x*-axis. ROC curves, like precision-recall curves, can also be used to assess different trade-offs for comparing the method outcomes. The ROC curve depicts relative trade-offs between benefits (TP rate) and costs (FP rate); the number of correctly classified, positive examples can be increased while decreasing additional false positives.

Table 9 illustrates the algorithms of the traditional methods in the performance evaluation. Each performance evaluation of the traditional algorithms was tested using Weka 3.7.1, which is a popular data mining tool that can be obtained from Java application programs. To avoid both overfitting and local optimization issues, the parameter values are not optimized but set as default value in Weka 3.7.1 as shown in Table 9.

## 5. Results

The results of the analysis for *stress* and *depression* are shown in Tables 10 and 11, respectively. First, according to the results using the proposed method (hybrid reasoning) for *stress*, the predictive value of a positive test (PPV), which is the probability of the correct result if a person has a *SC stress* and the result of the reasoning is *SC stress*, is 90.9%, and the predictive value of a negative test (NPV), which is the probability of the correct result if a person has no *SC stress* and the result of the reasoning is not *SC stress*, is 92.6%. PPV has four times greater performance than other methods, and NPV does not differ from the other methods. Second, according to the results of the proposed method for *depression*, PPV is 60.0% and NPV is 94.0%. PPV is therefore approximately two times better in terms of performance than other methods (Logistic and Ripper), and NPV is not significantly different from other methods. Because of the data imbalance, the results obtained using overall accuracy, precision, and recall are no different from other methods. For overall accuracy, the proposed method performed significantly better than other methods, taking into account the generalization between the importance of PPV and the importance of NPV as 1:1 (e.g., the simple average). The proposed method is one of the best reasoning methods because the reasoning method of PPV is more significant than the reasoning methods of NPV in this dataset obtained from a wellness service.

**Figure 2:**
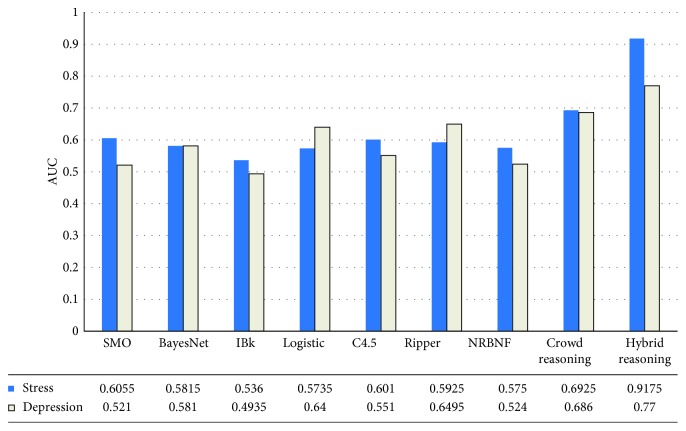
Performance comparison by AUC.

Figure 2 illustrates the results of comparative performance in a dataset with class imbalance, measured by AUC. The proposed hybrid reasoning method performed significantly better than the other algorithms. The results suggest that the hybrid reasoning method is superior to any conventional classification algorithms. The proposed method is the most suitable algorithm for classification of data in which class imbalance may be problematic, such as those aiding medical practitioners in making decisions about psychological well-being.

## 6. Discussion

### 6.1. Contributions

In this paper, a hybrid method, combining collective knowledge and crowd knowledge for diagnosing wellness in patients, was studied empirically, using a dataset with a high degree of class imbalance (i.e., actual healthcare data). The proposed method contributes to academia as well as practitioners.

First, we show that collective knowledge and crowd knowledge are complementary. The results suggest that the proposed method partially remediates the class imbalance problem. Hybrid reasoning with crowd knowledge extracted from open source data (i.e., a Google search) and collective knowledge (i.e., CBR) results in better performance than traditional methods (e.g., SMO, BayesNet, IBk, Logistic, C4.5, and crowd reasoning) as indicated by two measures, ROC and AUC, where accuracy is not an appropriate measure for class imbalance situations. This superior performance of the proposed method results from the complementary nature of these different kinds of knowledge.

Second, the results also suggest that big data, available online, and open source data may be used to improve the performance of reasoning systems. We used a Google search to compute the frequency of the association of two concepts in crowdsourced knowledge. The open data provided by a Google search is big data, as it results in a very large dataset with an unstructured format and can be updated in near real time.

Third, we found a cost-effective method for data-driven public health management in a smart city, as using open source data is easy to implement and less costly than reasoning-based methods from experts. For example, datasets with class imbalance are associated with cost-sensitive learning using conventional methods. By contrast, the proposed method is more economically efficient and has fewer maintenance requirements. Updating crowd knowledge and collecting open data are done automatically through a simple frequency search because open data owners such as Google, Facebook, and YouTube follow the common Web 2.0 strategy of letting users update their own data at virtually zero cost.

Last, the proposed method is extendable to other domains of a smart city in which class imbalance is problematic, such as prognosis and recommendation in various areas (e.g., medicine, planning, law, e-learning, knowledge management, and image processing). Further studies in different domains will make the proposed method more comprehensive.

Meanwhile, the proposed approach does not always produce the same result because the search result may vary depends on the time of searching for words. For example, search results from a search engine a year ago may differ from the current search results. After storing a lot of external knowledge on the web, analysis will provide consistent results. However, collecting a lot of external knowledge on the web using technique such as scrapping and then analyzing or predicting user's stress level or depression level could need amount of cost of time and effort. Therefore, the proposed method can have weakness in the consistency of result, but it can be an advantage in terms of efficiency. We confirmed that the proposed method is superior to other machine learning algorithms.

### 6.2. Concluding Remarks

As the fourth industrial revolution progresses, various personalized wellness services to be provided to citizens of each smart city will progress with big data collected from sensor network and intelligent analysis. These big data originate from either collective data or crowdsourced data, or both. Because data quality is crucial for the acceptance of big data analytics in the organizations [56], the issues associated with the class imbalance problem, resulting in lower data analysis performance, need to be resolved [57, 58]. Specifically, with regard to health data from a smart city, noise, uncertainty, and bias of crowdsourced data have to be addressed. Our research shows that crowd knowledge may be successfully combined with collective knowledge to enhance reasoning performance. Future research is needed to capitalize on the collaboration between conventional reasoning methods, and use of big data.

## Figures and Tables

**Table 1:** Co-occurring words for *depression*.

Word	Depression	Normal depression	Serious depression
Temperature	4,870,000	876,157	3,340,792
Humidity	1,590,000	424,202	662,866
Noise	4,120,000	842	1,892,800
Illumination	138,000	26,554	143,341
Anger	1,470,000	122,198	2,899,37
Stress	2,450,000	3,995	7,359,688
Fatigue	1,060,000	1,817	4,209,870
Fat	643,000	639	6,018,812
Diabetes	3,790,000	722,403	4,987,286
Heart disease	920,000	209	4,101
Hyperlipidemia	28,100	34,113	29,947
Cancer	1,500,000	1,942,493	145,190
Smoking	202,000	555,492	1,751,117
Drinking	328,000	796,695	4,630,933
Indigestion	1,100,000	500	7,544
Insomnia	205,000	867	896,134
Cold	925,000	122,044	75,463
Allergy	2,190,000	424,296	3,129,584
Neurosis	2,400,000	67	1,901
Blood pressure	6,630,000	734	2,788,095
Blood sugar	3,700,000	792,222	880,241
Wellness	21,600,000	71,406	5,309,378
Age	2,730,000	10,044	320,237
Gender	248,000	736	1,599,652
Weight	3,420,000	486,809	1,465,803
Solitude	837,000	108093	409,730
Hobby	903,000	4411	107,178
Yellow dust	775,000	321	730
Inconvenience	606,000	267279	706,899

**Table 2:** Association level for each word.

Word	Normal depression	Serious depression
Temperature	0.0005	0.0140
Humidity	0.0060	0.0065
Noise	0.0003	**0.0108**
Illumination	0.0034	0.0022
Anger	**0.0350**	0.0006
Stress	0.0277	**0.0455**
Fatigue	0.0309	**0.0329**
Fat	0.0064	**0.1097**
Diabetes	0.0148	**0.3164**
Heart disease	0.0001	0.0001
Hyperlipidemia	0.0014	0.0005
Cancer	0.0195	0.0002
Smoking	0.0032	**0.0169**
Drinking	0.0246	**0.0641**
Indigestion	0.0002	0.0001
Insomnia	0.0033	**0.0169**
Cold	**0.0268**	0.0002
Allergy	0.0005	**0.1639**
Neurosis	0.0000	0.0000
Blood pressure	0.0006	**0.1176**
Blood sugar	0.0060	0.0162
Wellness	0.0280	**0.0329**
Age	**0.0241**	0.0003
Gender	**0.0089**	0.0070
Weight	0.0324	0.0120
Solitude	0.0039	0.0054
Hobby	**0.0156**	0.0001
Yellow dust	**0.0006**	0.0000
Inconvenience	0.0024	0.0086

*Note*. Values in boldface indicate that the corresponding associated word is found more frequently. The meaning of these associated words helps to determine the depression level (normal or serious). For example, the high value for the associated word *noise* indicates that the depression level is likely to be more serious than normal.

**Table 3:** Profiles of respondents (*N*=334).

Category	Demographic	Frequency	Percentage (%)
Age group	20s	66	19.8
	30s	65	19.5
	40s	66	19.8
	50s	67	20.1
	≥60	70	21.0

Gender	Male	165	49.4
	Female	169	50.6

Location	Urban area	185	55.4
	Rural area	149	44.6

**Table 4:** Dataset and level of class imbalance.

	Mean (standard deviation)	Min (max)	Dataset	Level of class imbalance
			Samples ∗ attributes	Major : Minor
Stress	4.26 (0.847)	1.60 (6.65)	334 ∗ 51	10.7 : 1
Depression	3.05 (1.083)	1.00 (6.50)	334 ∗ 51	16.9 : 1

*Note*. The level of class imbalance indicates the ratio of the number of cases in the majority class compared to that of the minority class.

**Table 5:** Survey questionnaires.

Category	Items	Scales
Context	What is your context currently?	
	The current temperature is pleasant.	7 scales (1: strongly disagree, 7: strongly agree)
	The current humidity is pleasant.	7 scales (1: strongly disagree, 7: strongly agree)
	It is very quiet now.	7 scales (1: strongly disagree, 7: strongly agree)
	I had a lot of physical activity today.	7 scales (1: strongly disagree, 7: strongly agree)
	The current brightness is adequate.	7 scales (1: strongly disagree, 7: strongly agree)
	There is a lot of yellow dust around me.	7 scales (1: strongly disagree, 7: strongly agree)

Emotional status	How about your current emotional status?	
	I am very angry now.	7 scales (1: strongly disagree, 7: strongly agree)
	I am very stressed now.	7 scales (1: strongly disagree, 7: strongly agree)
	I am very depressed now.	7 scales (1: strongly disagree, 7: strongly agree)
	I am very tired now.	7 scales (1: strongly disagree, 7: strongly agree)
	I am happy now.	7 scales (1: strongly disagree, 7: strongly agree)

Diseases	Do you currently have any of the following symptoms or illnesses? If so, how serious is it?	
	Diabetes	7 scales (1: not at all, 7: very serious)
	Fat	7 scales (1: not at all, 7: very serious)
	Heart disease	7 scales (1: not at all, 7: very serious)
	Hyperlipidemia	7 scales (1: not at all, 7: very serious)
	Cancer	7 scales (1: not at all, 7: very serious)
	Smoking addiction	7 scales (1: not at all, 7: very serious)
	Alcohol addiction	7 scales (1: not at all, 7: very serious)
	Indigestion	7 scales (1: not at all, 7: very serious)
	Insomnia	7 scales (1: not at all, 7: very serious)
	Cold	7 scales (1: not at all, 7: very serious)
	Allergy	7 scales (1: not at all, 7: very serious)
	Neurosis	7 scales (1: not at all, 7: very serious)
	Blood pressure	7 scales (1: not at all, 7: very serious)
	Blood sugar	7 scales (1: not at all, 7: very serious)

Profile	General demographic questions for the survey	
	Gender	Nominal (1: male, 2: female)
	Age	Numeric
	Weight	Numeric
	Solitude	Nominal (1: alone, 2: with family, 3: with friend, 4: others)
	Hobby	Nominal (1: have regular hobbies, 2: have irregular hobbies, 3: no hobbies, 4: others)
	Location	Nominal (1: urban area, 2: rural area)

**Table 6:** Raw data samples from survey (100 samples out of 334).

No	P_GEN	P_AGE	P_AG	P_WEI	P_SOL	P_HOB	P_LOC	C_TEM	C_HUM	C_NOI	C_ACT	C_ILL	C_YD	D_FAT	D_DIA	D_HTD	D_HL	D_CAN	D_SMK	D_ACL	D_INDI	D_DEP	D_INS	D_COLD	D_ALG	D_NEU	D_BP	D_BS	E_ANG	E_STR	E_DEP	E_FTG
1	2	62	6	59	2	2	1	3	3	3	3	3	4	4	4	4	4	4	4	4	4	4	4	4	4	4	4	4	3	3	3	3
2	2	20	2	50	3	1	1	3	2	4	1	4	4	4	1	1	1	1	1	1	5	2	3	1	1	1	4	1	3	3	5	5
3	1	59	5	62	2	1	1	7	7	7	7	7	7	3	1	1	1	1	1	1	1	1	1	1	1	1	1	1	1	1	1	1
4	1	48	4	50	2	3	12	4	4	4	3	4	4	2	2	2	2	2	1	1	5	3	1	1	3	3	1	2	2	4	3	2
5	1	50	5	70	2	1	1	7	6	6	5	6	6	7	6	5	6	7	6	5	6	7	6	6	5	6	6	7	7	6	7	6
6	2	32	3	47	2	1	1	5	5	4	4	4	5	5	1	1	4	1	1	1	5	4	4	3	4	4	4	1	1	2	2	3
7	1	58	5	71	2	3	8	4	4	4	4	4	1	6	3	4	4	1	1	1	5	4	4	5	5	6	6	5	4	5	5	5
8	2	53	5	71	2	3	9	3	3	6	6	6	4	6	5	5	5	2	1	1	6	2	1	1	6	6	6	5	2	2	2	3
9	1	41	4	65	2	1	8	5	5	5	6	5	5	2	2	1	5	1	1	2	3	2	2	2	2	2	4	2	2	2	2	4
10	2	65	6	72	2	1	12	6	6	6	5	6	5	5	5	2	5	1	1	1	3	2	2	2	5	2	6	5	1	1	1	2
11	2	54	5	45	2	1	5	6	5	5	5	5	5	2	5	2	2	2	1	1	2	2	2	2	3	2	2	3	2	2	2	2
12	1	61	6	65	2	3	12	4	4	4	5	4	3	1	1	2	2	1	7	7	5	4	3	3	5	4	4	4	2	3	2	5
13	2	62	6	56	2	4	9	6	6	4	4	6	7	5	1	1	5	1	1	1	1	1	1	1	7	1	5	4	1	1	1	1
14	1	32	3	85	1	2	2	6	6	6	6	6	6	6	1	1	1	1	1	1	3	3	2	1	1	1	7	4	2	4	3	5
15	2	44	4	58	2	2	13	5	4	5	4	4	4	3	1	1	1	1	1	1	1	1	1	4	4	1	1	1	2	3	1	3
16	2	49	4	56	2	3	1	3	3	4	4	4	4	5	1	1	1	1	1	1	3	3	4	4	2	4	4	2	4	4	4	4
17	1	29	2	65	2	1	5	3	3	6	5	5	4	1	1	1	1	1	1	1	3	1	5	1	2	4	1	1	3	4	2	3
18	1	52	5	66	1	2	1	4	4	4	4	4	4	4	3	7	6	1	1	2	2	2	4	2	1	2	5	1	3	3	2	2
19	1	67	6	70	2	3	8	4	4	7	5	4	2	5	1	3	5	1	1	1	3	1	1	1	1	4	7	4	1	3	1	4
20	1	60	6	62	2	1	1	6	6	5	7	6	2	1	6	1	1	1	1	6	1	1	1	5	2	1	1	6	1	3	1	1
21	1	37	3	68	2	2	1	4	4	4	4	5	4	3	3	3	3	4	1	2	3	3	2	3	2	3	4	3	4	4	4	5
22	1	63	6	71	2	3	1	4	5	4	3	5	5	2	2	3	6	2	6	4	6	5	5	3	2	5	4	2	2	5	4	4
23	1	54	5	72	2	2	1	5	5	5	3	6	6	3	6	6	2	1	1	7	1	1	1	1	1	1	6	6	1	1	1	1
24	1	45	4	68	2	1	3	6	6	5	5	5	6	1	1	1	1	1	2	1	1	1	1	1	1	3	1	1	1	1	1	2
25	1	65	6	70	2	2	8	4	4	5	4	4	5	5	2	3	2	1	1	1	3	2	2	2	2	3	3	3	4	4	3	3
26	2	35	3	51	2	3	8	5	4	4	3	4	4	4	4	1	3	1	1	1	3	1	1	1	5	1	1	1	1	4	1	2
27	1	41	4	78	2	1	8	4	4	5	3	5	2	4	2	2	2	2	5	6	2	2	1	3	4	2	3	2	3	5	4	5
28	1	45	4	78	2	1	4	7	7	5	5	6	7	4	4	2	4	2	1	1	1	1	4	2	1	1	6	5	1	1	1	1
29	2	44	4	53	2	2	1	4	4	1	2	5	3	4	1	1	1	1	1	1	4	1	2	1	1	2	1	1	3	3	4	5
30	2	38	3	65	2	2	8	6	6	6	4	6	5	6	1	1	1	1	1	1	1	1	1	1	1	1	4	1	1	1	1	2
31	1	53	5	64	2	2	8	4	4	4	5	4	4	2	1	2	2	1	5	5	1	1	2	1	1	1	2	1	1	1	1	1
32	1	52	5	70	2	1	1	6	6	6	7	7	6	2	4	1	2	1	1	1	1	1	1	1	1	1	4	6	1	1	1	1
33	1	49	4	89	2	2	8	2	2	6	2	6	1	5	1	1	4	1	5	2	1	3	3	1	6	5	7	1	2	5	2	6
34	2	55	5	65	2	2	11	5	5	6	6	6	5	6	4	5	6	1	1	1	1	6	6	1	6	6	6	5	2	3	5	4
35	2	48	4	59	2	2	8	4	4	5	4	5	5	2	1	1	1	1	1	1	1	3	2	4	4	4	1	1	2	2	2	3
36	1	52	5	82	2	2	1	5	5	3	4	5	5	5	2	2	4	2	6	2	4	5	3	3	2	4	7	4	3	3	3	3
37	2	61	6	62	2	2	6	5	6	6	4	6	6	6	1	3	6	1	1	1	2	2	2	4	2	3	7	2	1	2	1	4
38	2	24	2	49	2	1	11	5	5	4	4	5	5	2	1	1	1	1	1	1	4	1	1	1	1	1	4	1	1	2	1	5
39	1	59	5	82	2	3	8	6	5	5	3	5	5	5	5	4	5	2	6	6	1	3	3	3	1	1	5	4	1	2	1	1
40	2	33	3	68	2	2	4	7	7	4	2	5	6	6	1	1	1	1	1	1	1	1	1	1	5	1	1	1	1	1	1	1
41	2	46	4	52	2	3	11	5	5	6	3	5	4	5	1	1	2	1	1	1	3	4	4	4	2	2	2	1	1	6	6	4
42	2	60	6	57	2	2	4	6	6	5	6	6	7	5	2	5	5	2	1	2	5	1	1	2	2	2	6	2	1	1	2	1
43	2	35	3	60	2	1	4	5	5	4	3	6	4	4	4	4	3	2	1	1	4	4	1	5	5	5	4	2	1	2	1	3
44	2	59	5	56	2	2	11	4	4	7	4	7	7	4	1	1	1	1	1	1	1	1	1	1	1	1	1	1	1	1	1	1
45	1	47	4	60	2	1	1	4	4	4	4	4	4	1	1	1	1	1	4	6	1	1	1	1	1	1	1	1	1	3	3	5
46	2	43	4	52	2	2	8	6	6	6	5	5	6	2	1	1	1	1	1	1	1	1	1	2	1	1	1	1	1	1	1	1
47	2	32	3	68	2	2	2	7	7	5	4	7	4	7	1	1	5	1	1	1	6	4	4	2	1	1	4	5	1	2	1	2
48	2	50	5	43	2	1	1	6	6	6	4	6	4	1	2	1	5	1	1	1	5	2	2	1	1	5	1	2	1	5	2	2
49	1	60	6	50	2	2	8	4	4	5	3	4	5	1	1	1	1	1	1	1	2	2	2	1	1	1	1	1	2	3	2	3
50	1	29	2	68	2	1	1	5	5	3	3	5	5	1	1	1	1	1	1	1	5	1	1	1	3	5	1	1	2	2	2	2
51	1	67	6	68	2	2	5	5	5	6	3	4	7	3	3	3	4	2	2	5	3	2	6	3	3	3	5	3	1	1	1	1
52	2	27	2	46	2	2	4	6	6	3	4	6	4	2	1	1	1	1	1	1	1	1	1	1	1	2	3	1	1	3	2	2
53	2	53	5	62	2	3	13	6	6	6	5	6	7	3	3	3	3	3	1	1	1	1	1	1	1	1	1	1	1	1	1	1
54	2	41	4	60	2	1	8	5	5	5	3	4	3	4	1	1	1	1	1	1	2	2	3	1	3	2	1	1	2	2	2	3
55	1	33	3	69	2	1	8	6	6	7	2	7	4	1	1	1	1	1	1	1	1	1	1	1	1	1	1	1	1	6	1	5
56	1	43	4	79	2	2	8	6	5	6	3	6	5	5	4	4	4	3	5	5	3	3	4	3	4	4	4	4	4	3	3	4
57	1	51	5	53	2	1	8	3	4	3	3	5	3	1	1	1	1	1	4	3	2	1	1	1	5	5	1	1	4	5	4	4
58	2	29	2	43	1	3	1	4	4	4	2	3	3	1	1	1	1	1	6	2	5	5	4	1	6	3	4	4	1	1	1	5
59	2	32	3	55	2	1	1	4	4	4	4	4	4	1	1	1	1	1	1	4	3	3	1	1	4	4	4	1	1	4	1	4
60	1	35	3	70	2	2	1	5	5	5	4	5	5	2	2	2	2	2	5	3	4	2	2	3	5	5	4	4	3	4	4	3
61	2	40	4	51	2	1	1	5	4	5	5	4	4	1	1	1	1	1	1	1	5	1	1	1	4	1	4	1	3	2	2	2
62	2	45	4	60	2	1	1	6	6	6	6	6	4	2	3	2	5	4	1	4	5	5	5	5	2	5	2	4	1	5	2	2
63	1	26	2	79	2	3	6	3	3	7	3	3	3	1	1	1	1	1	1	1	1	5	6	5	1	4	7	1	5	6	5	7
64	1	62	6	64	2	2	8	4	5	3	2	6	6	1	4	2	3	2	1	1	3	2	4	3	2	2	5	5	2	3	2	4
65	2	32	3	49	2	3	6	4	2	2	2	4	3	1	1	1	1	1	1	1	1	5	1	1	1	4	4	1	1	4	2	5
66	2	58	5	50	2	2	14	5	5	6	4	4	5	1	1	5	1	1	1	1	4	1	1	1	1	1	1	1	2	2	2	2
67	2	61	6	72	2	1	15	6	6	5	6	5	6	3	3	3	3	1	1	1	4	6	1	6	6	7	6	4	5	5	6	6
68	2	23	2	80	2	1	4	6	3	5	1	4	3	6	1	3	1	1	1	3	6	6	6	5	7	6	1	5	1	5	5	5
69	2	60	6	52	2	1	2	7	7	7	2	7	7	1	1	7	7	1	1	1	4	1	6	1	1	1	1	7	1	1	1	1
70	2	45	4	54	2	2	6	6	6	7	5	6	6	5	5	2	5	1	1	1	1	1	1	1	1	4	5	5	2	6	5	5
71	2	24	2	53	1	2	6	4	4	4	3	4	5	1	1	1	1	1	1	1	1	1	1	1	1	1	1	1	1	1	1	1
72	1	53	5	69	2	2	4	5	5	5	5	5	5	2	2	1	2	1	4	2	1	1	1	1	1	3	2	2	2	2	1	4
73	2	35	3	60	2	3	14	4	3	3	4	4	3	5	1	1	1	1	1	1	1	1	1	1	1	1	1	1	3	5	4	5
74	2	59	5	57	2	2	1	6	6	4	5	6	6	3	1	1	3	1	1	1	3	1	1	2	1	2	5	1	1	1	1	3
75	2	22	2	53	2	2	8	7	7	7	1	7	7	1	2	1	1	1	1	1	1	1	2	1	1	1	1	1	1	1	1	1
76	2	29	2	70	2	2	12	4	3	2	3	4	2	5	1	1	1	1	1	1	2	2	2	1	3	2	4	3	1	3	2	3
77	1	65	6	60	2	2	1	1	1	7	7	7	1	1	1	1	1	1	5	1	1	1	1	1	1	1	6	1	1	1	1	1
78	1	66	6	68	2	1	15	7	4	6	7	5	7	1	1	1	1	1	1	1	2	4	2	4	5	3	5	1	1	1	1	1
79	1	42	4	54	2	3	8	4	4	4	4	4	4	2	2	2	2	1	1	1	5	4	6	5	3	3	3	2	3	3	3	4
80	1	62	6	62	2	2	7	5	5	5	3	5	5	2	6	2	2	2	2	2	2	2	2	2	2	2	2	6	2	2	2	2
81	2	20	2	60	2	2	4	6	5	4	5	5	2	6	1	1	1	1	1	1	4	5	4	6	6	6	4	4	5	2	2	2
82	1	35	3	75	2	2	8	6	4	5	6	5	4	5	1	1	1	1	7	6	2	1	3	4	1	5	5	1	1	2	1	5
83	2	30	3	61	2	3	11	5	5	5	5	6	5	2	1	1	1	1	1	1	1	2	1	1	1	1	1	1	2	5	5	5
84	1	42	4	57	2	1	1	4	4	4	4	4	5	1	2	4	6	1	1	2	3	5	5	1	2	3	3	3	5	5	5	5
85	2	31	3	75	2	1	1	7	7	1	4	7	7	7	1	1	1	1	1	1	1	1	1	1	1	1	1	1	1	1	1	1
86	2	19	2	46	2	3	15	3	4	3	2	2	2	1	1	1	1	1	1	1	1	3	3	1	4	1	1	1	2	3	5	5
87	2	25	2	70	2	1	8	7	7	2	2	5	2	7	6	1	6	5	7	7	7	3	3	7	1	7	7	6	1	6	4	7
88	1	38	3	87	2	2	12	5	5	6	4	5	5	5	4	1	2	1	6	2	1	1	1	2	1	1	2	1	1	2	1	1
89	1	51	5	80	2	2	11	3	3	4	4	4	4	6	4	4	4	4	4	4	5	4	4	4	4	4	6	4	3	4	4	4
90	1	64	6	74	2	2	8	4	4	5	5	5	4	6	6	5	5	4	1	3	3	5	5	5	3	4	4	3	5	5	5	5
91	2	34	3	55	2	2	1	5	5	5	3	5	5	1	1	1	1	1	1	1	1	1	1	1	2	1	4	1	1	4	2	2
92	1	59	5	62	2	2	1	5	5	4	3	5	3	1	2	3	3	2	5	3	2	1	2	3	2	1	3	3	1	2	2	2
93	1	37	3	78	1	1	1	5	5	5	5	6	5	5	1	1	1	1	4	1	4	1	4	1	1	1	5	1	5	5	4	5
94	2	51	5	58	2	3	5	3	3	5	6	5	5	4	1	1	1	1	1	1	3	3	6	2	1	2	1	1	1	2	2	3
95	1	35	3	72	2	2	8	5	5	5	6	5	5	2	1	1	1	1	1	1	1	1	1	1	1	1	1	1	2	2	1	3
96	1	38	3	74	1	3	1	4	4	5	4	4	4	4	3	4	3	3	2	5	4	5	3	3	3	5	4	4	4	5	5	4
97	2	60	6	58	2	2	1	4	5	5	5	6	6	3	1	1	2	1	1	1	1	1	1	1	3	2	1	1	3	3	3	3
98	2	51	5	57	2	2	8	5	5	5	6	5	4	5	1	1	5	1	1	1	5	4	1	1	5	1	5	3	2	2	2	3
99	2	60	6	54	2	1	8	4	4	5	3	4	4	4	2	4	5	3	2	2	2	2	2	4	2	2	4	2	2	4	2	3
100	2	65	6	55	2	2	3	5	4	6	6	5	3	2	2	1	5	1	1	4	2	4	6	4	4	4	4	4	3	6	6	5

*Note*. P_Gen = gender, P_Age = age, P_AG = age group, P_WEI = weight, P_SOL = solitude, P_HOB = hobby, P_LOC = location, C_TEM = temperature, C_HUM = humidity, C_NOI = noise, C_ACT = activity, C_ILL = illumination, C_YD = yellow dust, D_FAT = fat, D_DIA = diabetes, D_HTD = heart disease, D_HL = hyperlipidemia, D_CAN = cancer, D_SMK = smoking addiction, D_ACL = alcohol addiction, D_INDI = indigestion, D_DEP = depression, D_INS = insomnia, D_COLD = cold, D_ALG = allergy, D_NEU = neurosis, D_BP = blood pressure, D_BS = blood sugar, E_ANG = current anger level, E_STR = current stress level, E_DEP = current depression level, and E_FTG = current fatigue level.

**Table 7:** Samples of comparing scores before and after categorization.

Stress (before)	Stress (after)	Depression (before)	Depression (after)
3	Normal	3	Normal
3	Normal	5	Normal
1	Normal	1	Normal
4	Normal	3	Normal
6	Abnormal	7	Abnormal
2	Normal	2	Normal
5	Normal	5	Normal
2	Normal	2	Normal
2	Normal	2	Normal

**Table 8:** Confusion matrix.

		Prediction	
		Positive	Negative
Real	Positive	TP (true positive)	FN (false negative)
	Negative	FP (false positive)	TN (true negative)

**Table 9:** Conventional algorithms considered in the experiment for performance comparison.

Algorithms	Options (for Weka)	Value
SMO	The complexity constant *C*	1
	Number of folds	5
	Kernel type	PolyKernel
	The epsilon for round-off error	1.0*E*−12
	Tolerance	0.0010

BayesNet	Search algorithm	K2
	Maximum number of parents	2
	Score type	Entropy
	Estimate algorithm	Simpler estimator
	Estimate algorithm option	1.0

IBk	Number of nearest neighbors (k)	1
	Nearest neighbor search algorithm	LinearNNSearch

Logistic	The ridge in the log-likelihood	1.0*E*−8 (default)
	The maximum number of iterations	−1

C4.5	Pruned/unpruned decision tree	Using unpruned tree
	Minimum number of instances per leaf	2
	Seed for random data shuffling	1

Ripper	Number of folds for REP	3
	Minimal weights of instances within a split	2.0
	Whether not to use pruning	Using pruning

NRBNF	Number of clusters to generate	2
	Maximum number of iterations for the logistic regression	−1
	Minimum standard deviation for the cluster	0.1

**Table 10:** Performance with stress dataset.

Classifier	Class	Overall accuracy	TP rate	FP rate	Precision	Recall
SMO	Normal	93.4132	0.984	0.773	0.948	0.984
	Serious		0.227	0.016	0.500	0.227
	Overall		0.934	0.723	0.918	0.934
	Overall (norm)		0.606	0.395	0.724	0.606

BayesNet	Normal	92.8144	0.981	0.818	0.944	0.981
	Serious		0.182	0.019	0.400	0.182
	Overall		0.928	0.766	0.909	0.928
	Overall (norm)		0.582	0.419	0.672	0.582

IBk	Normal	92.2156	0.981	0.909	0.939	0.981
	Serious		0.091	0.019	0.250	0.091
	Overall		0.922	0.850	0.893	0.922
	Overall (norm)		0.536	0.464	0.595	0.536

Logistic	Normal	87.4251	0.920	0.773	0.944	0.920
	Serious		0.227	0.080	0.167	0.227
	Overall		0.874	0.727	0.893	0.874
	Overall (norm)		0.574	0.427	0.556	0.574

C4.5	Normal	88.6228	0.929	0.727	0.948	0.929
	Serious		0.273	0.071	0.214	0.273
	Overall		0.886	0.684	0.899	0.886
	Overall (norm)		0.601	0.399	0.581	0.601

Ripper	Normal	91.018	0.958	0.773	0.946	0.958
	Serious		0.227	0.042	0.278	0.227
	Overall		0.910	0.725	0.902	0.910
	Overall (norm)		0.593	0.408	0.612	0.593

NRBNF	Normal	91.6168	0.968	0.818	0.944	0.968
	Serious		0.182	0.032	0.286	0.182
	Overall		0.916	0.766	0.900	0.916
	Overall (norm)		0.575	0.425	0.615	0.575

Crowd reasoning	Normal	89.8204	0.930	0.545	0.960	0.930
	Serious		0.455	0.070	0.545	0.455
	Overall		0.900	0.515	0.934	0.900
	Overall (norm)		0.693	0.308	0.753	0.693

Hybrid reasoning	Normal	92.5150	0.926	0.091	0.993	0.926
	Serious		0.909	0.074	0.465	0.909
	Overall		0.924	0.090	0.906	0.924
	Overall (norm)		0.918	0.083	0.729	0.918

**Table 11:** Performance with depression dataset.

Classifier	Class	Overall accuracy	TP rate	FP rate	Precision	Recall
SMO	Normal	93.4132	0.975	0.933	0.957	0.975
	Serious		0.067	0.025	0.111	0.067
	Overall		0.934	0.893	0.919	0.934
	Overall (norm)		0.521	0.479	0.534	0.521

BayesNet	Normal	92.8144	0.962	0.800	0.962	0.962
	Serious		0.200	0.038	0.200	0.200
	Overall		0.928	0.766	0.928	0.928
	Overall (norm)		0.581	0.419	0.581	0.581

IBk	Normal	94.3114	0.987	1.000	0.955	0.987
	Serious		0.000	0.013	0.000	0.000
	Overall		0.943	0.956	0.912	0.943
	Overall (norm)		0.494	0.507	0.478	0.494

Logistic	Normal	91.9162	0.947	0.667	0.968	0.947
	Serious		0.333	0.053	0.227	0.333
	Overall		0.919	0.639	0.935	0.919
	Overall (norm)		0.640	0.360	0.598	0.640

C4.5	Normal	93.1138	0.969	0.867	0.960	0.969
	Serious		0.133	0.031	0.167	0.133
	Overall		0.931	0.829	0.924	0.931
	Overall (norm)		0.551	0.449	0.564	0.551

Ripper	Normal	93.7126	0.966	0.667	0.969	0.966
	Serious		0.333	0.034	0.313	0.333
	Overall		0.937	0.638	0.939	0.937
	Overall (norm)		0.650	0.350	0.641	0.650

NRBNF	Normal	94.0120	0.981	0.933	0.957	0.981
	Serious		0.067	0.019	0.143	0.067
	Overall		0.940	0.892	0.921	0.940
	Overall (norm)		0.524	0.476	0.550	0.524

Crowd reasoning	Normal	94.6108	0.972	0.600	0.972	0.972
	Serious		0.400	0.028	0.600	0.400
	Overall		0.936	0.564	0.949	0.936
	Overall (norm)		0.686	0.314	0.786	0.686

Hybrid reasoning	Normal	92.5150	0.940	0.400	0.980	0.940
	Serious		0.600	0.060	0.400	0.600
	Overall		0.919	0.378	0.944	0.919
	Overall (norm)		0.770	0.230	0.690	0.770
